# Targeted Knockout of *CYP79A1* Reduces Cyanogenic Potential in Grain Sorghum

**DOI:** 10.1111/pbi.70746

**Published:** 2026-08-31

**Authors:** Evan D. Groover, Jianqiang Shen, Kiflom Aregawi, Sophie Li, Shahar Schwartz, Brian J. Staskawicz, Peggy G. Lemaux, David F. Savage

**Affiliations:** ^1^ Innovative Genomics Institute University of California Berkeley California USA; ^2^ Department of Plant and Microbial Biology University of California Berkeley California USA; ^3^ Department of Molecular and Cellular Biology University of California Berkeley California USA; ^4^ Howard Hughes Medical Institute University of California Berkeley California USA

*Sorghum bicolor* is a versatile and climate‐hardy C4 grass grown globally for food, forage and bioenergy. In sub‐Saharan Africa and South Asia, the expansion of sorghum‐based agriculture is closely linked to rural food security and income growth, particularly in drought‐prone regions. Despite its importance to smallholder livelihoods, broader adoption of sorghum is constrained by the accumulation of the cyanogenic glucoside dhurrin, a potent anti‐herbivory metabolite that is rapidly hydrolyzed to toxic hydrogen cyanide (HCN), causing tissue damage upon ingestion by grazing animals (Gleadow and Møller 2014). Dhurrin accumulates to particularly high levels in juvenile tissues (Ohio State University Extension 2021), which are commonly consumed by animals, and its presence in both grain and forage sorghum presents an adoption barrier for many smallholder and subsistence farmers.

Dhurrin biosynthesis is mediated by a metabolon comprising two cytochrome P450 enzymes, CYP79A1 and CYP71E1, which sequentially convert L‐tyrosine to *p*‐hydroxymandelonitrile, and the UDP‐dependent glucosyltransferase UGT85B1, which stabilizes this otherwise labile cyanohydrin through glucosylation to form dhurrin (Figure 1a) (Laursen et al. 2016). Studies using chemically mutagenized sorghum lines have established essential roles for each enzyme in this pathway. Loss‐of‐function mutations in *UGT85B1* cause severe pleiotropic defects consistent with cyanohydrin‐mediated autotoxicity (Blomstedt et al. 2016). In contrast, missense or nonsense mutations in *CYP79A1* abolish dhurrin production with only modest impacts on early growth and minimal effects on biomass accumulation or agronomic performance in mature plants, consistent with the proposed role of dhurrin as a transient nitrogen reservoir in developing seedlings (Blomstedt et al. 2012; Sohail et al. 2020). To date, deployment of *CYP79A1* mutant alleles has been limited to a small number of forage and grain sorghum backgrounds. Because elite grain sorghum cultivars can retain substantial cyanogenic potential, reducing cyanogenic potential in grain sorghum addresses a persistent constraint in mixed crop‐livestock systems. Here, we establish a genome‐editing strategy to reduce or eliminate cyanogenesis in the grain sorghum inbred RTx430 by targeting *CYP79A1*.

**FIGURE 1:**
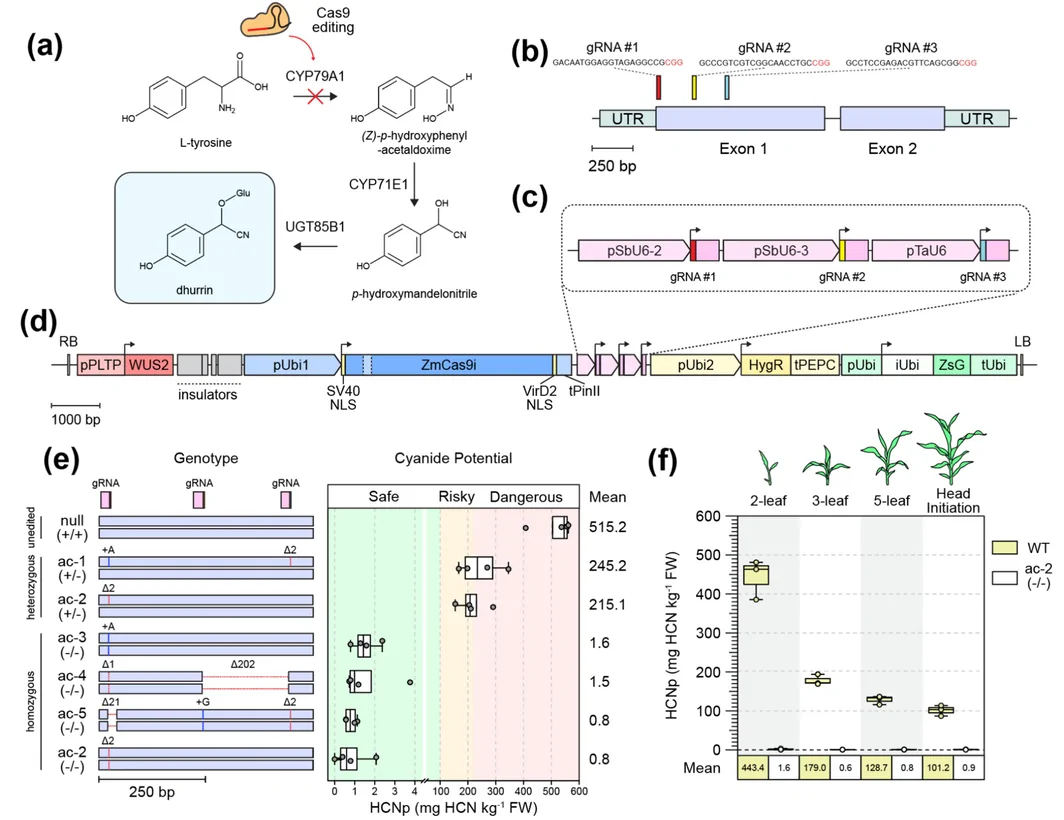
Cas9‐mediated editing of *CYP79A1* abolishes sorghum cyanogenicity. (a) Enzymatic pathway for dhurrin biosynthesis from L‐tyrosine. *CYP79A1* catalyzes the first committed step and was targeted for Cas9‐mediated genome editing. (b) Gene model of *CYP79A1* showing untranslated regions (UTRs; green) and exons (purple). Three guide RNAs (gRNA #1–3) targeting exon 1 are indicated; protospacer sequences are shown in black with the adjacent PAM motif (NGG) highlighted in red. (c) gRNA expression cassettes used for multiplex editing. gRNA #1 is driven by the sorghum *U6‐2* promoter, gRNA #2 by the sorghum *U6‐3* promoter, and gRNA #3 by the wheat *U6* promoter. (d) T‐DNA architecture of the binary vector used for transformation. The T‐DNA contains a maize codon‐optimized Cas9 (ZmCas9i) with two nuclear localization signals (NLS), three guide RNA (gRNA) expression cassettes, a hygromycin resistance marker (hygromycin B phosphotransferase, HygR), a ZsGreen1 fluorescent reporter (ZsG) and the morphogene *WUSCHEL2* (*WUS2*). ZmCas9i is driven by the maize ubiquitin 1 promoter (pUbi1), HygR by the switchgrass ubiquitin 2 promoter (pUbi2), and ZsGreen1 by the sorghum ubiquitin promoter (pUbi). Right and left T‐DNA borders (RB and LB) delineate the transferred region. (e) Genotypes and cyanogenic phenotype of stable T_1_ lines. Left, haplotype‐resolved edited alleles for each line across the targeted region of *CYP79A1*, annotated as heterozygous (+/−) or homozygous (−/−) for edits; an unedited T_1_ control is shown (null, +/+). Right, cyanide potential (HCNp; mg HCN kg^−1^ fresh weight, FW) measured from *n* = 4 biological replicates per genotype. Background shading indicates livestock grazing risk thresholds (green, safe; orange, risky; red, dangerous) (Ohio State University Extension 2021). Boxplots show the median (center line) and interquartile range (IQR) (box); whiskers extend to the most extreme values within 1.5 × the IQR, and points represent individual biological replicates (outliers beyond the whiskers, if present, are shown as points). The mean HCNp for each genotype is reported at right. (f) Developmental dependence of cyanogenic potential in wild‐type (WT) and homozygous *cyp79a1* knockout plants from the ac‐2 line (e). HCNp was measured at the indicated developmental stages (2‐leaf, 3‐leaf, 5‐leaf and head initiation) from most recently expanded leaves. Boxplots are defined as in (e) with *n* = 3 biological replicates per genotype; mean values are shown below each group.

Using the pGL222 binary T‐DNA system described previously (Shen et al. 2025), we targeted the first exon of *CYP79A1* using three evenly spaced guide RNAs (Figure 1b) to increase the likelihood of producing both small insertion‐deletions (indels) as well as larger deletions spanning multiple cut sites. Guide RNAs were selected with high predicted on‐target activity and minimal off‐target potential (Methods). Guide RNAs were expressed monocistronically under distinct monocot U6 promoters (Figure 1c) together with an intronized, maize‐codon‐optimized Cas9. The T‐DNA also contained a hygromycin resistance marker for tissue culture selection, the morphogenic gene *WUSCHEL2* to enhance transformation efficiency (Aregawi et al. 2022; Shen et al. 2025), and the fluorescent marker *ZsGreen1* to facilitate identification of transgenic and transgene‐free segregants (Figure 1d).

The final T‐DNA construct was transformed into a total of 205 sorghum RTx430 immature embryos (IEs) across two independent rounds of transformation (Aregawi et al. 2022). After initial transformation, IEs were recovered in co‐cultivation medium for 6 days at 28°C, transferred to resting media for one week at 28°C, moved to embryo maturation media (EMM) for 4–8 weeks with hygromycin selection, and finally transferred to rooting media without selection for root establishment. After two weeks in hygromycin selection in embryo maturation media, explants had an 80.5% embryo transformation efficiency as assessed by fluorescence microscopy (Figure S1a).

A subset of 42 regenerated explants were transferred to soil as independent transformants, 40 of which (95.2%) flowered and produced seed (Figure S1b). All explants were sequenced to confirm transgene integration and screened for *CYP79A1* editing. Of these, 30 T_0_ transformants showed detectable signals of editing in the seventh fully expanded leaf as assessed by Sanger sequencing using Inference of CRISPR Edits (ICE) analysis, while 12 showed no editing signal (Figure S1c). Among reproductively viable edited transformants, efficiencies ranging from 14 to 100% of edited alleles were observed (likely resulting from a heterogeneous mixture of edited and unedited alleles within a leaf sample), with an average editing efficiency of 70.46% (Figure S1c). To study the effect of chimeric editing on T_0_ cyanogenesis, a subset of 13 mature T_0_ plants were screened for their cyanogenic potential (HCNp) by a modified picrate assay (Bradbury et al. 1999), amended with a dhurrin‐hydrolyzing enzyme buffer (Gleadow et al. 2012), performed on the seventh fully expanded leaf at maturity. We observed that the CRISPR knockout score was strongly negatively correlated (Pearson's *r* = −0.942; *R* ^2^ = 0.887) with cyanide production (Figure S1d), indicating that successful editing is quantitatively linked to the depletion of foliar cyanogenic potential. T_0_ transformants with high (> 90%) knockout scores were advanced to T_1_, where segregants were screened for absence of ZsGreen1 fluorescence, absence of PCR‐amplifiable T‐DNA region and presence of on‐target edits. Multiple independent edited, T‐DNA‐free lines were recovered and advanced for subsequent phenotypic and genotypic analysis.

Genotyped T_1_ plants representing five independent edited backgrounds were screened for cyanogenic potential as homozygotes, heterozygotes or both and compared to an unedited null T_1_ control (Figure 1e). Plants were assayed at the two‐leaf seedling stage, when dhurrin accumulation is near maximal. Homozygous *cyp79a1* knockouts were negligibly cyanogenic (0.8–1.6 mg HCN kg^−1^ fresh weight), whereas heterozygous plants exhibited approximately half the cyanogenic potential of unedited nulls (Figure 1e). Variation among heterozygous T_1_ individuals likely reflects partial CYP79A1 dosage, but might also be influenced by minor variability in assay performance or tissue sampling. Consistent with established livestock grazing guidelines (Ohio State University Extension 2021), only homozygous knockouts fell below thresholds considered hazardous for incidental grazing. Depletion of cyanogenic potential was stable across early vegetative development, and a representative homozygous knockout line maintained negligible cyanogenic capacity relative to wild‐type controls throughout this period (Figure 1f).

From nine T_0_ transformants exhibiting > 90% editing, a total of 14 edited alleles (and two wild‐type nulls) were recovered as homozygous T_1_ lines (Figure S2a). Based on these outcomes, guide RNA #1 was the most active of the three guides tested, accounting for 88.9% of all edited T_1_ haplotypes and present in all recovered edited alleles (Figure S2b). Given its high efficiency and conserved sequence within the early coding region of *CYP79A1* exon 1, this guide represents a strong candidate for deployment in additional sorghum cultivars. As potential off‐target edits were not systematically evaluated in this study, further segregation and breeding may be required to separate unintended edits from the desired *CYP79A1* alleles.

Together, these results demonstrate that *CYP79A1* knockout can effectively ablate cyanogenic potential in grain sorghum across a physiologically relevant developmental window, supporting its utility as a practical and heritable genome‐editing target for reducing cyanogenic risk in elite grain sorghum cultivars.

## Funding

This work was supported by Chan Zuckerberg Initiative (CZIF2022‐007203). D.F.S. is an Investigator of the Howard Hughes Medical Institute.

## Conflicts of Interest

D.F.S. is a co‐founder and scientific advisory board member of Scribe Therapeutics. B.J.S. is a scientific co‐founder of and serves on the board of directors for Mendel Biotechnology. He also serves on the scientific advisory boards of Verinomics and the Sainsbury Laboratory. WUSCHEL2 genes were used for research purposes, and commercial applications for their use requires a paid non‐exclusive license from Corteva Agriscience. Manuscript authors have no relationship with Corteva Agriscience.

## Supporting information


**Figure S1:** Transformation, genotyping and screening of T_0_ *CYP79A1* mutants.
**Figure S2:** Heritable editing outcomes of *CYP79A1* mutants.

## Data Availability

The data that supports the findings of this study are available in the Supporting Information of this article.
