# Supplementary material for: Targeted Knockout of CYP79A1 Reduces Cyanogenic Potential in Grain Sorghum
Source: Plant Biotechnol J. 2026 Aug 31;24(11):5865–7. doi: 10.1111/pbi.70746 (PMC13530436; doi:10.1111/pbi.70746)
Supplement: Supplementary file 1 [file PBI-24-5865-s001.docx]

**Supplementary Figure 1. Transformation, genotyping, and screening of T_0_ *CYP79A1* mutants.**

**(a)** Summary of embryo transformation efficiency across two independent rounds of *Sorghum bicolor* RTx430 immature embryo transformation. Embryo transformation percentage was calculated as the number of independent fluorescent regenerants (not counting subcultures) exhibiting stable fluorescent marker expression at four weeks post-transformation divided by the total number of embryos inoculated. Of 165 fluorescent embryos, 42 independent transformants were transferred to soil and advanced to greenhouse growth.

**(b)** Recovery and preliminary genotyping outcomes for T_0_ sorghum transformants assessed for *CYP79A1* editing in the seventh fully expanded leaf. Editing status was determined by Sanger sequencing across the *CYP79A1* target region. “Edited” (blue) indicates detection of one or more indels within the target window; “Unedited” (green) indicates only wild-type sequence was detected; “Edited, aborted” (grey) indicates transformants that did not survive to flowering and could not be phenotyped further.

**(c)** Editing efficiencies of all edited T_0_ lines from **(b)**, quantified by Sanger sequencing and ICE analysis. Editing efficiency represents the inferred fraction of edited alleles per line, measured as editing score in ICE. Each point corresponds to a single edited T_0_ event (n = 28). Median (solid black line) and mean (red dashed line) values are indicated.

**(d)** Relationship between inferred knockout score derived from ICE analysis and cyanogenic potential in T_0_ plants. Cyanogenic potential (HCNp; mg HCN kg⁻¹ FW) was measured from the seventh fully expanded leaf of mature plants and plotted against the knockout score, defined as the inferred fraction of alleles containing frameshift mutations or deletions >21 bp. Each point represents an individual T_0_ plant (*n* = 13). The solid line indicates a least-squares linear regression; coefficient of determination (r²) is shown.

**Supplementary Figure 2. Heritable editing outcomes of *CYP79A1* mutants.**

**(a)** Haplotype-resolved *CYP79A1* alleles recovered from homozygous edited T_1_ plants. For each T_1_ individual, both alleles are shown schematically, with deletions (Δ), insertions (+), and substitutions annotated relative to the wild-type sequence. Red dashed lines indicate large deletions spanning the multiple guide RNA (gRNA) target sites, which are indicated by pink boxes. Individual alleles were PCR-amplified and sequenced by Sanger sequencing from T_1_ segregants. Vertical bars group lines derived from a common edited T_0_ parent, and line identifiers at left indicate individuals selected for downstream phenotypic analyses (Fig. 1). Summary matrices at right indicate the specific allelic outcomes observed at each gRNA target site for each line.

**(b)** Inheritance frequency of edits at each gRNA target site across T1 lines. Bars indicate the percentage of genotyped T1 lines carrying at least one edited allele at the corresponding gRNA target site, calculated across all homozygous edited T1 individuals.

**Methods**

**Plant material and growth conditions**

Seeds of *Sorghum bicolor* genotype RTx430 were obtained from the Germplasm Resources Information Network (GRIN, USDA-ARS; PI 655998). T_0_ transformant plants were cultivated in 3-gallon pots containing SuperSoil (Rod McLellan Company) in the UC Berkeley Oxford Research Unit South Greenhouse under controlled conditions of 30°C/24°C (day/night) with a 16-hour light/8-hour dark photoperiod. T_1_ plants were cultivated in 3.5-inch pots in a growth chamber at 28°C, 100 μmol photons m⁻² s⁻¹ light intensity, a 16-hour light/8-hour dark photoperiod, and 80% relative humidity.

**gRNA and CRISPR Construct Creation**

Candidate target sequences within *CYP79A1* were identified in the *S. bicolor* cv. RTx430 v2.1 genome (Phytozome; https://phytozome-next.jgi.doe.gov/info/SbicolorRTx430_v2_1). Three guide RNAs targeting the first exon of *CYP79A1* (SbiRTX430.01G012200) were designed using the Benchling CRISPR tool. Guides were selected based on high predicted on-target activity and low predicted off-target potential. Guide RNAs were spaced approximately 200 bp apart within the coding sequence to increase the likelihood of large (hundreds of bases), loss-of-function deletions between cut sites. Targeted regions were PCR-amplified from genomic DNA and verified by Sanger sequencing prior to cloning. Notably, guide RNAs #1 and #3 have no predicted complete genomic off-target sites in the sorghum pangenome, but guide RNA #2 has one homologous off-target in some sorghum cultivars in another cytochrome P450 gene (SbiRTX430.10G183100), which is predicted to act orthogonally in phenylacetaldoxime biosynthesis. Although prospective off-target sequences were not sequenced in this study, targeted or whole-genome sequencing to detect genomic off-target mutations should be performed for regulatory assessment and field deployment. Notably, *cyp79a1* mutant lines displayed growth rates and overall growth habits indistinguishable from unedited control plants.

Guide RNAs were cloned into the pGL222 binary vector using a modified shuttle vector system described previously [(Shen *et al.*, 2025)](https://paperpile.com/c/OQQIBF/0Ep0). Guide RNA spacers were introduced into a modified pGL198 shuttle vector (Addgene #180472) containing a tandem array of monocot U6 promoters (Sorghum *U6.2.3*, Sorghum *U6.3.1*, and *Triticum aestivum* U6), each monocistronically driving a guide RNA scaffold. The shuttle vector also contained an intronized maize codon-optimized Cas9 driven by the maize ubiquitin-1 promoter and terminated by the potato PINII terminator. Spacer sequences were introduced by Gibson Assembly (NEBuilder HiFi DNA Assembly Master Mix, NEB E2621L). Shuttle vectors were transformed into chemically competent TOP10 *E. coli* and fully sequenced to confirm assembly. Verified shuttle vectors were recombined into pGL222 using Gateway LR Clonase II (Invitrogen 11791020), and final constructs were sequence-verified.

***Agrobacterium*-mediated transformation**

Binary constructs were introduced into *Agrobacterium tumefaciens* strain LBA4404 Thy⁻ by electroporation. Transformants were selected on YEP medium supplemented with 100 mg L⁻¹ thymidine, 50 mg L⁻¹ gentamicin, and 50 mg L⁻¹ spectinomycin. Immature embryos were harvested 12–14 days post-anthesis and co-cultivated with *Agrobacterium* suspension (OD₅₅₀ = 0.7) prepared in PHI-I medium supplemented with 0.005% (v/v) Silwet L-77 and 0.2 mM acetosyringone. Embryos were co-cultivated at 28°C for one week, transferred to resting medium for one week at 28°C in the dark, and then placed on embryo maturation medium containing 15 mg L⁻¹ hygromycin for 4–6 weeks until regeneration. Regenerants were transferred to rooting medium and subsequently to soil. All media conditions are as described in [(Aregawi *et al.*, 2022; Shen *et al.*, 2025)](https://paperpile.com/c/OQQIBF/xbg3+0Ep0).

**Visualization of T-DNA integration**

T-DNA integration and segregation were monitored using the ZsGreen1 fluorescent reporter. Fluorescence was assessed weekly using a Zeiss SteREO Lumar.V12 epifluorescence stereomicroscope equipped with a QImaging Retiga SRV camera. Embryo transformation efficiency was calculated as the number of independent fluorescent regenerants (not counting subcultures, i.e. where more than one transformant came from a single embryo) divided by the total number of embryos inoculated.

**DNA Extraction**

Genomic DNA was extracted using a Chelex-based protocol. Leaf tissue (~1 cm²) was homogenized in 280 μL of 10% (w/v) Chelex® 200–400 mesh resin (Bio-Rad 95621) prepared in 5 µM Tris-HCl (pH 8.0). Samples were vortexed, incubated at 95°C for 5 min, vortexed again, and centrifuged at maximum speed for 1 min. Supernatant was used directly for PCR amplification.

**Genotyping transformants and quantifying editing**

T-DNA presence was assessed using primers spanning a 1.5-kb region within the T-DNA region and starting immediately downstream of the T-DNA left border. *CYP79A1* edits were genotyped using primers amplifying a ~1 kb region encompassing all three guide RNA target sites. Amplicons were Sanger sequenced, and editing outcomes were quantified using EditCo ICE analysis (ice.editco.bio). ‘Editing efficiency’ refers to the percentage of alleles containing any guide-proximal edit, whereas ‘knockout score’ refers to alleles containing frameshift mutations or deletions >21 bp. T_1_ seedlings were screened for fluorescence using a Leica M205C stereomicroscope, and non-fluorescent plants were genotyped using leaf punches collected at the time of cyanogenic analysis.

**Cyanide quantification**

Cyanogenic potential was quantified using a modified sodium picrate assay [(Bradbury *et al.*, 1999)](https://paperpile.com/c/OQQIBF/vOnp) optimized for complete enzymatic hydrolysis of dhurrin. Foliar cyanogenic glucoside detection kits were obtained from the Australian National University (Kit E;<https://biology.anu.edu.au/research/divisions/konzo-prevention-unit/kits-posters>) and adapted for quantitative analysis using a β-glucosidase degradation buffer commonly employed for dhurrin hydrolysis [(Gleadow *et al.*, 2012)](https://paperpile.com/c/OQQIBF/KnJ2b).

Mid-leaf tissue was harvested from the second, third, fifth, or seventh fully expanded leaf (i.e., no further blade elongation) prior to the emergence of the subsequent leaf. For all comparisons, plants were sampled based on matched developmental stage (leaf number and expansion status), such that knockout and control plants were assayed at equivalent physiological stages, even when this required sampling at slightly different chronological ages. Leaf tissue was sliced into ~2 mm strips using a safety razor and thoroughly ground with a mortar and pestle until complete homogenization was achieved. All tissue processing steps were optimized to ensure maximal cyanide release within the reaction vessel, and mechanical disruption was performed as rapidly as possible to minimize premature volatilization or loss of hydrolyzed cyanide prior to measurement.

For each sample, 100 mg of homogenized fresh tissue was weighed and transferred to a sealed reaction vial containing 1 mL of β-glucosidase degradation buffer composed of β-glucosidase (2 mg mL⁻¹; Sigma G0395) dissolved in 0.1 M trisodium citrate buffer (pH ~5.5). A sodium picrate indicator strip was suspended within each vial. Negative control vials lacking tissue and positive control vials supplied with the kit were included in all assays. β-glucosidase degradation buffer was prepared fresh for each experiment. Sealed vials were incubated for 24 h at room temperature in the dark to allow complete enzymatic hydrolysis of dhurrin and absorption of released hydrogen cyanide by the indicator strip.

Following incubation, indicator strips were removed and eluted in 5 mL of ddH₂O in 15-mL culture tubes. Tubes were incubated for 30 min, including 10 min of agitation at 40 rpm on a rotary shaker. Eluates were mixed by pipetting, and absorbance was measured at 510 nm using an Ultrospec 3000 UV/Visible spectrophotometer (Pharmacia Biotech). Absorbance values were normalized to negative controls and multiplied by a factor of 396 to calculate cyanogenic potential, expressed as mg HCN kg⁻¹ fresh weight.

Assay accuracy was verified in all experiments using kit-provided positive controls and potassium cyanide (KCN) standards spanning a range of concentrations. For T_1_ phenotyping experiments, leaf tissue from three to four independent plants per genotype was analyzed. For T_0_ phenotyping, samples were obtained non-destructively by collecting leaf hole punches from the seventh fully expanded leaf following transfer to the greenhouse. For all phenotyping, non-transformed (T_0_ and T_1_) or unedited (T_1_) negative control plants were measured in parallel.
